# Free text adversity statements as part of a contextualised admissions process: a qualitative analysis

**DOI:** 10.1186/s12909-018-1158-6

**Published:** 2018-04-02

**Authors:** Lysa E. Owen, Stephanie Ann Anderson, Johnathan S. Dowell

**Affiliations:** 1 University of Dundee School of Medicine, Angus, Dundee, UK; 2 Dundee Institute for Healthcare Simulation, University of Dundee, Ninewells Hospital and Medical School, Dundee, DD1 9SY UK

**Keywords:** Medical education, Academic performance, Selection, Admission criteria, Widening access, Widening participation, Diversity, Socio-economic status

## Abstract

**Background:**

Medical schools globally are encouraged to widen access and participation for students from less privileged backgrounds. Many strategies have been implemented to address this inequality, but much still needs to be done to ensure fair access for all. In the literature, adverse circumstances include financial issues, poor educational experience and lack of professional-status parents. In order to take account of adverse circumstances faced by applicants, The University of Dundee School of Medicine offers applicants the opportunity to report circumstances which may have resulted in disadvantage. Applicants do this by completing a free text statement, known as an ‘adversity statement’, in addition to the other application information. This study analysed adversity statements submitted by applicants during two admissions cycles. Analysis of content and theme was done to identify the information applicants wished to be taken into consideration, and what range of adverse circumstances individuals reported.

**Methods:**

This study used a qualitative approach with thematic analysis to categorise the adversity statements. The data was initially analysed to create a coding framework which was then applied to the whole data set. Each coded segment was then analysed for heterogeneity and homogeneity, segments merged into generated themes, or to create sub-themes.

**Results:**

The data set comprised a total of 384 adversity statements. These showed a wide range of detail involving family, personal health, education and living circumstances. Some circumstances, such as geographical location, have been identified and explored in previous research, while others, such as long term health conditions, have had less attention in the literature. The degree of impact, the length of statement and degree of detail, demonstrated wide variation between submissions.

**Conclusions:**

This study adds to the debate on best practice in contextual admissions and raises awareness of the range of circumstances and impact applicants wish to be considered. The themes which emerged from the data included family, school, personal health, and geographical location issues. Descriptions of the degree of impact that an adverse circumstance had on educational or other attainment was found to vary substantially from statements indicating minor, impact through to circumstances stated as causing major impact.

## Background

Equality and fairness in medical school admissions are ethical imperatives. The purpose of medical education is to graduate individuals with the attributes, knowledge and skills to meet the present and future healthcare needs of society. It is argued that this is best achieved if students are representative of the general population, and have a good understanding of the healthcare needs, barriers and the social determinants of health within the particular populations they serve [1]. The United Kingdom (UK) and Scottish Governments, British Medical Association (BMA) and Medical Schools Council (MSC) have made recommendations on how institutions can address inequality and enhance access for under-represented groups [2–6].

In spite of these drivers and benefits of enhancing diversity, there is evidence that studying medicine remains elitist [2, 7, 8] and simple solutions remain elusive. The Oxford dictionary defines adversity as “a difficult or unpleasant situation” [9]. A range of factors have been identified as adverse in terms of medical school admission and career progression. These include racial, gender and socioeconomic factors, financial hardship and educational disadvantage [1, 2, 4, 10–20].

Those applicants with such barriers are less likely to apply to study medicine, and if they do apply, are less likely to be successful in their application; in some contexts it has been recorded that 80% of medical applicants originate from only 20% of schools or colleges [4] and applicants with professional parents make up 84% of applications [21]. Additionally, higher education can seem culturally foreign to individuals who have experienced such adversity [13], as it is dominated by those from the highest socio-economic groups and professional backgrounds [11, 21]. It has been suggested that medical school admissions criteria such as high academic attainment, participation in work experience, evidence of voluntary and extra-curricular activities can result in bias towards those from more privileged backgrounds, as the ability to access such opportunities can depend on schooling, demographics, socio-economic status and parental factors. However, although disadvantaged students are less likely to apply and less likely to be successful, research demonstrates that when successful they can outperform their peers as they progress through their university education [6, 12, 22]. Increasing diversity at medical school has been reported to help students to be better prepared to meet the needs of the public after graduation, and has the potential to improve workforce recruitment to areas and specialities where there are current shortages [23, 24]. For example, those areas where general practitioner (GP) recruitment is most challenging tend to be the same areas which are currently under- represented in medical school admissions. Widening access and participation can also benefit individual students in terms of enhancing social mobility as well as benefiting the educational environment for all students [25].

Despite emerging evidence that increasing diversity, by recruitment of more non-traditional students, positively influences medical undergraduate programmes, there is still a relative paucity of evidence addressing the question “What can be done to effectively enhance widening access and participation (WAP)?” It appears likely that multiple strategies are needed to achieve the aims of genuine diversity, as has been achieved, for instance, in terms of gender.

Recommendations for best practice, in WAP, have been made [26–28] and some examples of initiatives to enhance WAP are briefly outlined here. One UK medical school has designed a course specifically for students from areas of high socio-economic deprivation [1], other schools adjust the entry requirements from eligible applicants, accepting reduced grades from applicants from low achieving schools, so-called ‘Contextual Adjustment’ (CA) [12]. Others have described a combination of strategies including schools outreach and enhanced support programmes, alongside the approaches mentioned above [1, 29]. There has also been helpful work identifying which selection methods offer the best evidence for fairness in admission processes [18, 30]. These include the use of multiple mini interviews (MMI) and aptitude testing such as the United Kingdom Clinical Aptitude Test (UKCAT), The Biomedical Admissions Test (BMAT), The Medical College Admissions Test (MCAT) and The Graduate Australian Medical School Admissions Test (GAMSAT) within application processes [8, 21, 31–37] . The use of such aptitude testing has been shown to be both reliable and valid, and longer term data on predictive validity are now emerging [38]. Their impact on WAP, though positive, appears marginal and is contested. Research has demonstrated both the potential that additional testing may have on enhancing diversity [8], and also, the slow rate of progress being made [39]. This highlights the challenge of incorporating methods to enhance diversity amongst medical students, and the need more research into fair admissions processes.

Many medical schools operate contextualised admissions process [40]. Contextual admissions use “a range of factors, including comparative school and socio- economic data, to establish ‘relative’ achievement of applicants which may allow Higher Education Institutions to identify the individuals ‘real’ potential to achieve” [12]. They aim to ensure “holistic assessment” of candidates while incorporating the philosophies of fair admissions [40]. There is however, little literature demonstrating what contextual data is collected, how different institutions collect such data, and how the data is used within selection processes. This research offers an original contribution by analysing the range of adverse circumstances, that applicants to our medical school asked to be considered as part of the selection process. This approach offers an additional potential mechanism, alongside other strategies, to improve equity and therefore selection practice but also creates challenges. This paper will focus on analysing the types of statements applicants made and does not consider issues of verification or probity [41]. As admissions processes vary between institutions, regions and countries, it is beyond the scope of this paper to make specific recommendations for other contexts, but rather, highlights the potential that the use of adversity statements offer to this important area.

In summary, the current literature highlights the need for widening access initiatives to provide a diverse workforce to meet population health needs. Research about how this can be achieved is emerging and this study contributes to this area by revealing the wide range of adverse circumstances applicants report. This will be of interest to institutions as they develop robust and fair, contextualised selection processes.

### Context

This research was carried out at The University of Dundee, School of Medicine (UDSM). It is located in Dundee, Scotland and has approximately 800 undergraduate medical students [42]. In a typical year, around 1500 applications are received, and around 300 offers are made to successful applicants. Throughout the United Kingdom all university applications are made through a single centralised service; The Universities and Colleges Admission Service (UCAS). Applicants can apply for multiple courses and universities using this system but limits them to four medical school applications per yearly cycle. The application includes personal details, including post-code, and school attended (for applicants still in school at the time of application), academic credentials (qualifications gained or expected) and a personal statement outlining their personal interests, motivation for applying for their chosen course and demonstrating personal attributes which make them suited to a particular course of study.

In addition to the electronic application process, through UCAS, as outlined above, UDSM include the following invitation: *“We would like to give you the opportunity to provide any additional details relating to adversity that you think may be relevant to your application by completing an on-line questionnaire form. Your response will be held in strict confidence, in accordance with the Data Protection Act 1998, and will only be used in the assessment of your application.”* Having accessed a web link, applicants are then invited to complete a free text box that invites applicants to document “*any special circumstances that you think we should consider as part of your application. Specifically, we invite you to tell us if there are adverse circumstances or factors beyond your control that are not conducive to study or which have otherwise hindered your application. Your response will be held in strict confidence. Please note that false reporting may invalidate your UCAS application. If there are adverse circumstances or factors beyond your control that are not conducive to study or which have otherwise hindered your application please provide details in the box below”* [24]. The responses are used as part of a contextualised admissions process. Handling the very wide range circumstances that applicants may face, the qualitative nature, and subjectivity of the responses, may be challenging for institutions operating contextual admissions processes. This paper presents the results of a thematic analysis of the adversity statements submitted to this institution over two admissions cycles.

## Methods

The University of Dundee Research Ethics Committee (UREC) granted approval for this study. All adversity statements submitted during 2012 and 2014 admission cycles were included. Two admissions cycles were selected in order to collate sufficient quantity of data, and two non-consecutive cycles were chosen to reduce the likelihood of including re-application from the same applicants. All applicants had been informed that their anonymised data may be used for research purposes [24]. The research team included one current medical student (SA), hence initial anonymization of all data was performed by a staff researcher (LO) who is also a practising clinician. Anonymization included removing dates of events, names, and any personal identifiable information. This included generalising some descriptions of medical conditions or personal circumstances where their unusual nature could have led to identification. There were no exclusion criteria.

A descriptive qualitative approach, set within a realistic paradigm using thematic analysis was used. This approach was selected as it offered a methodology which could identify common threads without loss of complexity, but did not require the very high level interpretive complexity associated with grounded theory or hermeneutics [43]. This method has been described by Braun and Clarke [44] as a suitable method for identifying, analysing and reporting patterns (themes) within data, and this research used the phases of thematic analysis as a guide. A qualitative analysis software package (Atlas.ti®) was used as a repository for the data and to assist with the organisation and analysis of the data [45, 46]. Firstly, familiarisation took place: two researchers (SA, LO) independently gained a sense of the data by reading several times, made notes and observations about the data independently and then came together to reach agreement on definitions and the strategy for coding. Codes are researcher generated constructs symbolizing and attributing meaning to individual datum to seek patterns, create categories and subsequently theorising. The code is an attempt to capture the primary content or essence of an extract of data [47]. Generating the coding framework continued until no new additional codes were generated. Once the coding framework was agreed one researcher then applied the codes to the entire data set. While analysing, the researchers looked for heterogeneity and homogeneity within the data.. Codes with high degrees of homogeneity were then collected and grouped together as themes. The researchers were then able to refine the themes by iteratively comparing, reorganising, and seeking alignment with codes and their definitions. Where heterogeneity was identified, this allowed the identification of subthemes. Analysis was completed when both researchers were in agreement that no further new codes were identified and no further iterations were indicated.

## Results

In the two admissions cycles studied, a total of 3633 applications were received, and of these, 384 applications included submission of an adversity statement. This equates to 10.6% of applications.

**Fig. 1:**
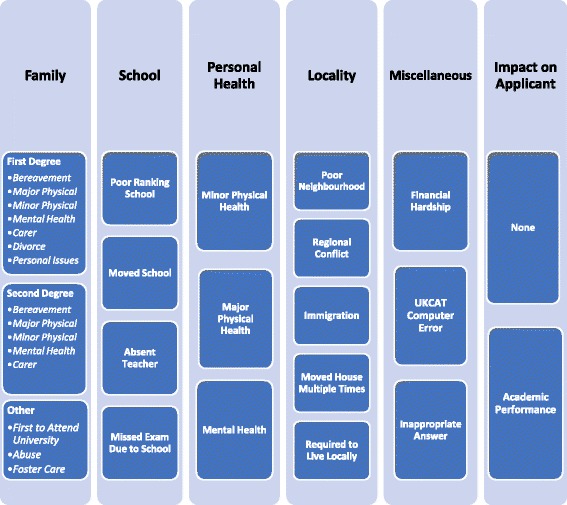
Themes, sub-themes and clusters

Using non-consecutive admissions cycles 2 years apart, rather than consecutive cycles, reduced the likelihood of re-applications being considered. No identical adversity statements were identified and it is assumed that the data originated from 384 individual applicants. The statements ranged in length from 2 to 1637 words. The major themes, sub-themes and clusters that emerged from analysis of adversity statements are shown in Fig. 1. Table 1 ranks the adverse circumstances by frequency of reporting.

**Table 1:** Frequency of adverse events reported

Theme	Subtheme	Cluster	Number of times theme arose
Family	First Degree	Carer	76
		Major Medical	63
		Bereavement	52
		Minor Medical	52
		Divorce	51
		Mental Health	49
		Personal Issues (not specified)	1
	Second Degree	Bereavement	58
		Carer	29
		Major Medical	21
		Minor Health	12
		Mental Health	11
	Other	First to attend University	6
		Abuse	5
		Foster Care	2
School	Moved School	–	12
	Poor Ranking School	–	6
	Absent Teacher	–	5
	Missed Exam -School Error in Timetable	–	1
Personal	Minor Medical Condition	–	51
	Major Medical Condition	–	41
	Mental Health Condition	–	36
Location	Immigration	–	15
	Required to Live Locally	–	11
	Regional Conflict	–	8
	Moved House Multiple Times	–	5
	Poor Neighbourhood	–	1
Miscellaneous	Financial Hardship	–	29
	Inappropriate Answer	–	9
	UKCAT Computer Error	–	1
Effect on Applicant	Academic Performance	–	128
	None	–	14

The major themes were family issues, personal health issues, personal living circumstances and school or education issues, and finally the degree of impact described by applicants.

The first major theme was family issues such as bereavement, major health problems, mental health problems, caring responsibilities, divorce, abuse, foster care, and being ‘first in family’ to progress to university education.

Many respondents reported bereavements. Some applicants used detailed narratives to describe their closeness to their late relative, type of illness, duration and impact of treatments, subsequent caring responsibilities and educational impact of the time of death, and their experience of the bereavement,

Others made simple statements of fact, such as *“My [parent] passed away in [year].”*

Statements regarding family issues were sub-divided into first degree and second degree family issues, where first degree includes parent, child or sibling, and second degree includes grandparents, aunts or uncles. Third degree relatives include cousins and great-grandparents. The bereavement experiences reported ranged from a first-degree relative’s death, close to the time of gaining formal qualifications, to second and even third degree relatives’ deaths some years ago. A large number of applicants reported family health conditions, with wide variation in the severity and longevity of the medical and mental health conditions reported, and the degree of impact reported by the applicant. For example, major physical or mental health issues included significant stroke with ongoing major functional impairment, ICU admission with sepsis, leukaemia, breast cancer, paranoid schizophrenia, whilst minor physical health conditions reported included hypothyroidism, glaucoma, and eczema. Some statements, for example stated mental illness, without further detail. A full list of the conditions reported is appended. Reported family health conditions included: 164 in a first-degree 44 in a second degree relative. There is a degree of overlap between an applicant having a relative with a long term health condition or disability and the role of carer. Caring for a family member as a result of their long term medical condition was reported 76 times for first degree and 29 for second degree relatives.

Some medical issues mentioned in the statements, had an indication of whether the circumstance was transient or long-term, others did not specify duration.

Divorce appeared in 13% of statements.

The second major theme was the applicant’s personal health status, where statements included major physical health problems (11% of respondents), minor physical health problems (51%) and specific or unspecified mental health problems (36%).

The third major theme was school related issues including poor ranking school with low academic standards, absent subject teacher, and circumstances resulting in frequent changes of school. Changing schools featured in 3% of statements.

The fourth major theme was related to personal living circumstances and included living in an area of socio-economic deprivation where high academic achievement was unusual, financial hardship, living in, or having relatives living in an area of political conflict, issues around immigration and visa status, and frequent house moves. A small number of additional items were grouped as miscellaneous and included technical problem with aptitude testing, time-table error for examinations diet. Nine inappropriate answers were identified which were not considered relevant to the selection process e.g. disputing a decision on fee status, or visa issues.

### Impact on applicant

In 33% of adversity statements the applicant described the negative impact of the circumstance on their educational attainment. However, 3% described *not* being affected by their adversity, instead describing their ability to overcome their adversity and their view that they had not been academically disadvantaged and had still achieved their full potential. The remainder of respondents having stated their adverse circumstance gave no indication of the impact on their academic or other attainment. The timing of the adverse event also varied from recent and during exam periods, to years in the past while growing up.

Having outlined the major themes identified in the data, the degree of impact reported by applicants is now presented. The portrayal of the impact of an adverse circumstance varied widely. Many applicants gave detailed accounts of the negative impact of their circumstances on their academic achievement or credentials, for example; *“this hugely distressing experience made curricular work even more challenging and the difficulties of managing school work with home life were made even more complex”.*

Conversely, some applicants reported that the circumstances did not have any impact on their performance or credentials; *“I continued to be committed to my school studies and did not allow it to blight my education”.*

If an applicant implied a circumstance affected them negatively, there was a wide range of detail of the degree of impact they experienced. For example, divorce was reported in 51 statements. Accounts of the impact ranged from one applicant stating; *“family separation”* whilst another detailed their circumstances to include nature and degree of marital dispute, legal consequences, impact on applicant’s responsibilities, mental health and education.

The frequency with which circumstances were reported was analysed and quantified using the groundedness measure tool in Atlas.ti®, and is shown in Table 1. Bereavement was reported 110 times, making this the most frequently reported circumstance, whilst living in an area of socio-economic deprivation being reported by only one applicant. The frequency of other circumstances can be seen in.

In summary, the analysis of 384 adversity statements revealed a wide range of personal health, family, educational, and community circumstances which applicants wished the medical school to consider. In addition, the range of severity was wide and the amount of detail about both the circumstances and their impact also varied widely. The discussion which follows will relate the circumstances revealed by this research to the literature, in order to encourage debate and discussion on how adjustments should be made in such cases.

## Discussion

This discussion will summarise and consider how the results contribute to research in the field, and situate our findings within the current literature. It will also consider the strengths and limitations of the work, the implications for policy and practice and propose future research work.

These results demonstrate that when applicants to medical school are invited to provide additional information on adverse circumstances that 10.6% make use of this opportunity to inform the institution of their circumstances. The types of issues revealed, the range of experiences and the perceived impact and degree of detail offered all show a very wide range, which would be expected as the range of life experiences, and their impact on individuals, is highly personal and varies widely. From the quantitative perspective, most of the adversity statements made related to bereavement, divorce and personal health. The open and qualitative format of the invitation to submit adversity statements would appear to offer an opportunity for applicants to reveal highly personal information, which the formal application process would be unlikely to identify. Many of the situations which would be considered as adverse, such as having a role as carer, or attending a low achieving school, living in an area of socio-economic deprivation, or being first in family to access higher education, were reported less frequently. These less frequently reported adverse circumstances represent less personal and subjective experiences and may also be more readily identified through other parts of the admission process and other metrics.

The way the identified themes in this research relate to the existing literature will be discussed in the following section. Firstly adversity in the area of family issues will include bereavement, caring role, divorce, and first in family status. Bereavement has been shown to have an impact on academic performance [48] but it can be challenging to quantify the degree of impact on any individual applicant. Academic qualifications awarding authorities such as the Scottish Qualifications Authority (SQA) have mitigating circumstances policies which allow a school to draw attention to the fact that a candidate has experienced issues, such as bereavement, around the time of their examination which is considered to have impacted on their performance, and allows the awarding authority an opportunity to compensate their grade. This highlights that, although the SQA acknowledge short term circumstances, such as a recent bereavement, many of the other adverse circumstances identified in this research reveal longer term disadvantage and may not be considered through such mitigating circumstances processes. Similarly, other exam boards have an emphasis on only acute issues being considered [48, 53]. English examination board ‘Edexel’ have a system where students with specific adversities are given an allowance of marks. The “most exceptional cases”, such as terminal illness of candidate or parent/carer is given 5%, and more “minor problems” such as headache, or noise during an exam more than momentary, is given 1% [48].

Discussion of applicants’ awareness of this process, and the interface between universities and qualification awarding authorities is beyond the scope of this paper but highlights the potential for ‘double counting’ of short term adversity. Those young people who have a significant burden of responsibility of caring for another family member because of medical, mental health or disability issues are considered to be educationally disadvantaged, and have lower academic grades as a consequence [49]. It is acknowledged that many young people perform these roles without being recognised. UDSM suggest caring roles as an example of adversity and invite young carers to disclose this using the adversity form [24]. Some applicants described ‘being carers’ as part of their adversity statement in relation to them participating in voluntary work with the elderly or disabled. The authors suggest that there can be misunderstanding around the term ‘carer’ and consider that this indicates the need for additional guidance for applicants to indicate the type of caring role, the duration, and the impact, to fully understand the degree of adversity experienced. Divorce also was indicated in adversity statements. Ham [50] demonstrated academic performance can be impacted with changes in family structure. To consider divorce in context, with over 9 thousand divorces being granted in Scotland between 2014 and 2015 [51] this poses a potential challenge in contextualised admissions. Some of the applicants who included divorce as an adverse circumstance also described additional impact such as abuse, restraining orders, frequent house moves and frequent changes of school; this was categorised separately.

The UDSM form [24] discusses adversity as a “special circumstance”, however, if a large proportion of applicants are affected, how this information is used in the selection process is challenging. The authors suggest that while the experience of divorce alone would not be considered adverse, with the opportunity that free text adversity statements offers, those who have experienced or witnessed associated abuse or mental health problems leading to significant disruption of home life and studies can have an opportunity to have this considered.

Personal health statements were common and included both short term and long term injuries or illness impacting on applicants’ education or examinations. Conditions were classified into minor and major physical conditions (by LO a practicing clinician). Although it has been shown that minor conditions such as rhinitis, and long-term conditions such as diabetes can have an effect on academic performance [19, 52] without a clear description of significant impact, making an adjustment would seem disproportionate. Qualifications awarding authorities, such as the SQA’s Results Services can take a “medical condition” or bereavement into consideration, but this is not expanded upon further in their guidance [53]. This is similar to the English Edexel’s “Special Circumstances” services which can take acute health issues into consideration, but long term conditions are excluded [48]. The authors suggest that institutions may need to work with awarding authorities in handling such information in contextual admissions. Although disabilities are manged through policies offering ‘reasonable adjustments’ the only disability disclosed in this data was dyslexia. Adjustment for this is made through the adjustment of additional time during aptitude testing in UKCAT. This may still leave some applicants with undisclosed disabilities at a disadvantage.

While bereavement and divorce were frequently reported, where there is limited evidence that these result in disadvantage in university admission, there were three areas which were under-reported, in spite of a strong evidence base that these are academically disadvantageous. These were first-in-family status, attending a poorly achieving school and living in an area of significant socio-economic deprivation. Few applicants reported that they were the first in their family to attend university. The literature suggests that individuals with professional parents, with tertiary level education are much more likely to apply, and be successful in gaining university places than those who are ‘first -in- family’ to access higher education [21]. It appears that although the absence of parents who understand and can support the process of university admissions is disadvantageous, few applicants used adversity statements to highlight this. Attending a poorly performing school also featured in very few statements. The literature is consistent in identifying poor educational experience as a barrier to progression to tertiary education, and especially medicine [4]. Only 1% of responses, in the two admissions cycles studied in this research, used the free text adversity statements to disclose attending a low progression rate school as an adverse factor. During the 2014 admission cycle we estimate that 14% of Scottish domiciled applicants under the age of 21 attended schools with low progression rates^1^. This would strongly suggest that this particular circumstance is under-reported by applicants using free text adversity statements.

The final area which appears to be under-reported in this research is home address. Only one applicant reported living in an area of socio-economic deprivation, even though this has been shown to result in educational disadvantage and many governments have a range of targets and initiatives to enhance the efforts of educational institutions to promote widening access for young people in such areas [54]. Many institutions use the postcode or area code of the applicant as a marker for deprivation. In many cases this data can be generated from the applicant’s address, and during the admission cycle included in this research there were applicants who lived in areas of deprivation, who did not include the information as an adversity statement. The opportunity of including this information as part of a free text adversity statement allows applicants to give details of impact of this deprivation on their personal attainment. There appears to be link between underreporting of first in family status, attending a low progression rate school and living in an area of socio-economic deprivation, suggesting these to be linked. Although the reasons applicants did not include this were not explored in this research, it raises the possibility that those very applicants who may benefit most from contextualised application process in this circumstance are unlikely to report this. The authors suggest that this may be due to lack of awareness amongst those applicants. Or alternatively, applicants might assume that the university already have this information through the other parts of the admissions process so does not need to be reiterated. A further possibility is that such applicants may be concerned about the possibility of stigmatisation or even fear discrimination if they revealed this information.

This should be addressed by enhanced outreach and communication efforts targeted at no-traditional potential applicants.

In summary the results of this research reveal a wide range of adverse circumstances and the degree of impact on academic attainment. Some adverse circumstances are frequently reported, but some which are known to be associated with educational disadvantage appear to be under-reported. The authors suggest that greater clarity regarding the type of information being sought and guidance to suggest that adverse circumstances can be more thoroughly evaluated when more detailed information about the impact of these circumstances is provided.

### Strengths and limitations of this research

This research is thought to be the first study where the content and themes of free text adversity statements as part of a contextual admission process have been analysed in detail, and hence offers an original contribution to the field. The authors acknowledge limitations of this work. This research analysed the circumstances which applicants chose to reveal to the institution using an online free text adversity statement. This research did not seek to verify the validity of the participant’s responses [55, 56]. Neither did the researchers match the data to other markers of adversity as part of a contextual admissions process. The authors did not collect data to find out *why* some adverse circumstances were included or omitted from applications.

This research was carried out using one institution’s adversity statements from two non-consecutive admission cycles, therefore, this study may not necessarily be generalizable to other contexts. The analysis was carried out retrospectively and so no additional data could be collected. Whilst qualitative research inherently involves interpretation, this variability was reduced by having two researchers classify the themes independently before negotiating a framework for analysis. Due to the relatively simple level of interpretation and abstraction required in classifying this data, a third researcher was not required. A further limitation was that, for ethical reasons, only the staff researcher (LO) read all the raw data in its original form before the anonymization process. This may have resulted in some loss of richness of the data, but was unavoidable for ethical reasons. A final limitation is that many young people with potential to become excellent doctors, but who have experienced adversity through no fault of their own, may not reach the application stage, and may not recognise their own circumstances as disadvantageous, thus may not allow an institution the opportunity to consider them. Another limitation is that some young people who have experienced significant adversity may choose not to disclose this as part of a psychological coping mechanism where they wish to achieve their ambitions on their merit alone, and may wish to ‘move on’ from their difficult circumstances. Anecdotally, we are aware of some successful applicants who are current students who did not use this opportunity to describe their adverse circumstances either because they had concerns it might disadvantage their application, or alternatively that they had determination to succeed on merit alone.

### Implications

The adversity statements studied included descriptions of longstanding significant challenging circumstances that reasonable judgement would suggest were likely to have a detrimental effect on some applicant’s ability to mount a competitive application, both in terms of academic attainment as well as difficulty with extracurricular activities and work experience. Enabling applicants to highlight such factors to improve equity would then seem to have face validity. Thus, we argue that the use of free text adversity statements could have an important role to play, alongside other strategies, as part of a contextual admissions process. The approach described here could be improved by additional guidance and the systematic inclusion of impact statements but this area requires further work. Similarly the use of contextual data requires more open discussion and exploration.

There are legitimate concerns regarding the verification of such information which is time consuming and sometimes could be considered excessively intrusive where the nature of adversity may be sensitive. Given existing concerns regarding the integrity of personal statements there is clearly a need to also address this. This paper has not discussed verification of adversity statements or made recommendations to other institutions on exactly how individual medical schools should use such data but suggest that these aspects should be studied in subsequent scholarly work on the advantages and disadvantages of such statements, and how the data can be best used in fair admission processes in the context of each institution.

Future research is planned to examine the relationship between free text adversity statements and other application data. The authors also plan to conduct research exploring reasons why some applicants who have experienced adverse circumstances might choose not to declare these during application. It would also be desirable to conduct similar research in a range of different contexts to enhance generalizability.

## Conclusion

This research thematically analysed applicant adversity statements from two admissions cycles. The major themes that emerged were family, school, personal health, locality, miscellaneous and impact on the applicant. Circumstances such as being the first to attend university, postcode and school adversities in medical school applications were described by applicants as disadvantageous, which is consistent with current literature [4, 13, 21]. This study also highlighted some other personal circumstances such as personal illness, family illness, bereavement and divorce, which applicants revealed as part of a contextual admission process. The impact of an adverse circumstance was also found to vary substantially from descriptions of minor to major impact. Although some of the circumstances which can disadvantage an applicant may be captured in other parts of admissions processes, many of the circumstances described, and the extent of impact on the applicants academic performance, would not otherwise have been identified through the existing admissions processes.

This study adds to the debate on best practice in contextual admissions. The researchers conclude that the opportunity for medical school applicants to provide free text adversity statements has an important role to play in a contextualized admission process and merits further study. This research raises awareness of the range of circumstances and impact applicants wish to be considered as part of robust, transparent admissions processes which enhance diversity and provide fair opportunities for all.

## Abbreviations

BMA: British Medical Association
BMAT: Biomedical admissions test
CA: Contextual adjustment
GAMSAT: Graduate Australian medical school admissions test
GP: General practitioner
MCAT: Medical college admissions test
MMI: Multiple mini interviews
MSC: Medical Schools Council
SQA: Scottish Qualifications Authority
UDSM: University of Dundee, School of Medicine
UK: United Kingdom
UKCAT: United Kingdom Clinical Aptitude Test
UREC: University of Dundee Research Ethics Committee
WAP: Widening Access and Participation

## Footnotes

1 We were only able to analyse the “last educational establishment” for Scottish under 21 applicants, not the whole body of applicants, so this figure is an approximation.
